# Modulation of HMGB1, PRF1, TRAIL, and M-CSF after TARGIT-IORT in breast-conserving surgery: defining a unique acute immune landscape

**DOI:** 10.1007/s12094-026-04353-1

**Published:** 2026-04-17

**Authors:** Yastira Ramdas, Catherine Worsley, Pieter Willem Meyer, Carol-Ann Benn

**Affiliations:** 1 Radiation Oncology, Corewell Health William Beaumont University, Royal Oak, 3601 W 13 Mile Rd, Royal Oak, MI 48073 USA; 2 Department of Immunology, University of Pretoria, Lynnwood Rd, Hatfield, Pretoria, 0002 South Africa; 3 National Health Laboratory, Sandrigham, Johannesburg 2017 South Africa; 4 The Breast Care Unit, NetCare Milpark, 9 Guild Road, Parktown, Johannesburg, 2000 South Africa

**Keywords:** Immunogenic cell death (ICD), Breast cancer, Radiation therapy, Radiotherapy boost, TARGIT-IORT (targeted intraoperative radiotherapy), Tumor microenvironment, Immunomodulation

## Abstract

**Background:**

Targeted intraoperative radiotherapy (TARGIT-IORT) delivers a single high-dose boost to the tumor bed during breast-conserving surgery (BCS) and is now recommended only within clinical trials or registries. Its acute systemic immunogenic effects relative to BCS-only are not well characterized.

**Methods:**

Women with histologically confirmed breast carcinoma were allocated in an alternating 1:1 sequence to BCS + TARGIT-IORT or BCS-only groups. Peripheral blood was sampled pre-operatively and 24 h post-surgery. ICD and cytokine/DAMP mediators, including HMGB1, PRF1, TRAIL, FASL-NFSF6, CASP-8, GZMB, CRT, TLR4, RAGE, GDF15, and M-CSF, were quantified using ELISA. Perioperative changes were analyzed using the Wilcoxon signed-rank test; between-group Δ differences were assessed using the Mann–Whitney *U* test.

**Results:**

Forty-two patients were enrolled (26 BCS + TARGIT-IORT; 16 BCS-only). TARGIT-IORT induced a distinct early immune shift across ICD markers. HMGB1 showed the most pronounced change (3410.5 → 359.8 pg/ml; *p* < 0.001), significantly greater than that after BCS-only (1040.7 → 263.6 pg/ml; Δ*p* < 0.001). TRAIL (246.1 → 155.5 pg/ml; *p* = 0.001) and FASL-NFSF6 (1087.1 → 839.3 pg/ml; *p* = 0.003) also declined significantly after TARGIT-IORT, whereas changes following BCS alone were modest. TARGIT-IORT increased M-CSF (266.7 → 386.7 pg/ml; *p* = 0.037) and reduced TLR4 (1456.3 → 1331.3 ng/ml; *p* = 0.007), with no significant perioperative changes in RAGE, GDF15, CRT, GZMB, or CASP-8.

**Discussion:**

Within 24 h, TARGIT-IORT generates a coordinated ICD-related and cytokine/DAMP signature, marked HMGB1 depletion, death ligand modulation, TLR4 engagement, and M-CSF upregulation, consistent with rapid immune priming. These findings are exploratory and should be interpreted within a translational framework. Larger, randomized multicentre studies with extended longitudinal and tissue-level immune profiling are needed to confirm these signals.

## Introduction

Supported by numerous randomized and meta-analysis, breast-conserving therapy (BCT) combined with external beam radiation therapy (EBRT) provides equivalent overall survival to mastectomy for stage I and II breast cancers, while significantly reducing the risk of local recurrence compared to lumpectomy alone [1]. Targeted intraoperative radiotherapy (TARGIT-IORT), which delivers a single large dose (20 Gy in 15 min) directly to the tumor bed at the time of lumpectomy, was an emerging alternative to EBRT for carefully selected women with early-stage breast cancer evidenced by trials such as TARGIT-A [2, 3]. The clinical role of IORT has markedly contracted and coupled with the emergence of ultrashort, highly effective external beam schedules such as Five Fraction Radiotherapy (FAST)-Forward have led the 2024 American Society for Radiation Oncology (ASTRO) guideline to restrict IORT to use only within prospective trials or registries [4].

IORT is administered immediately after tumor resection, coinciding with the wound-healing phase characterized by increased cytokine production and immune cell infiltration. This timing and single dose strength allows IORT to modulate the tumor microenvironment in ways that EBRT cannot [5, 6]. Studies show that IORT alters the local cytokine profile, increasing levels of immune-activating cytokines and reducing those that promote tumor proliferation and invasion [3]. The perioperative delivery of IORT occurs precisely when damage-associated molecular patterns (DAMP) release and innate immune activation are at their peak, positioning it as a potent initiator of immunogenic cell death (ICD) [7, 8]. High-mobility group box 1 (HMGB1) represents a central early DAMP in this cascade, while perforin-1 (PRF1), tumor necrosis factor-related apoptosis-inducing ligand (TRAIL), and Fas ligand (FasL) reflect downstream cytotoxic and death receptor pathways engaged during ICD [3, 8, 9]. Macrophage colony-stimulating factor (M-CSF) provides complementary insight into monocyte–macrophage recruitment during acute tissue injury.

Together, these markers capture key elements of ICD and wound-healing biology, making them well suited to detect the immediate systemic immune shifts uniquely elicited by TARGIT-IORT. This study examined the ICD-related and cytokine pathway response in breast cancer patients undergoing breast-conserving surgery (BCS) in two treatment arms, BCS + TARGIT-IORT and BCS-only. All patients, 6 weeks post-BCS, received an equivalent 50 Gy with EBRT, with the BCS-only group receiving an additional EBRT boost of 10 Gy in 5 fractions. Biomarkers were quantified from plasma collected pre-operatively, 1 h before BCS, and 24 h post-surgery. Given current guideline restrictions limiting IORT to clinical trials or registries, the immunological insights from this work should be interpreted within a translational framework rather than as evidence for therapeutic efficacy.

## Methods

### Study design and ethics

This prospective cross-sectional study was conducted at the Breast Care Unit of Netcare Milpark Hospital in Johannesburg, South Africa. Ethical approval was obtained from the Research Ethics Committee of the Faculty of Health Sciences, University of Pretoria (reference number 466/2021), and written informed consent was obtained from all participants.

### Eligibility of patients and patient assignment

This study enrolled adult female patients diagnosed with breast carcinoma who presented to the study site between September 2021 and July 2022. Most of the participants were from South Africa or neighboring countries.

**Fig. 1:**
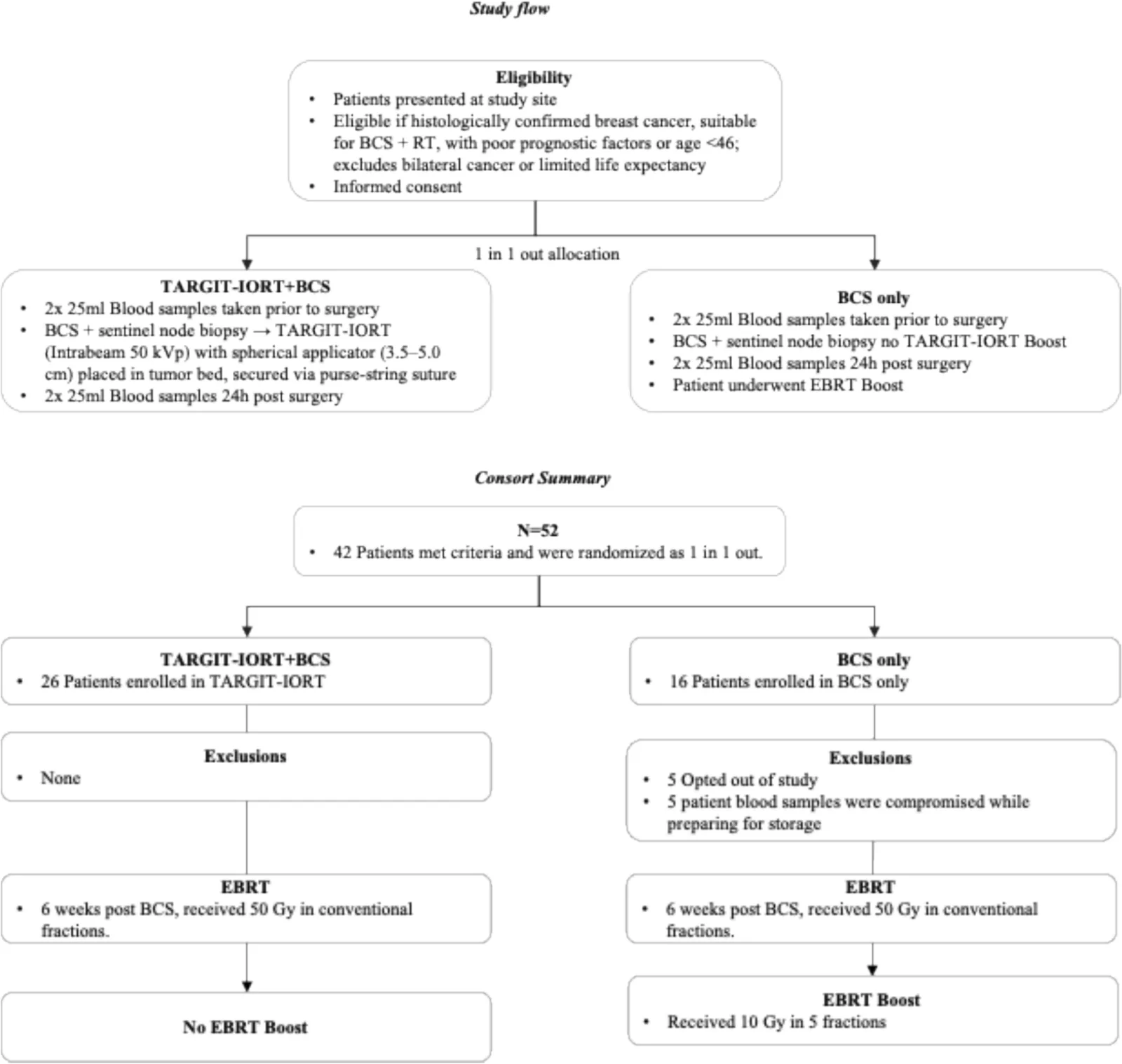
Study flow and consort diagram. BCS, breast-conserving surgery; RT, radiotherapy; TARGIT-IORT, targeted intraoperative radiotherapy; EBRT, external beam radiotherapy; SNB, sentinel node biopsy; kVp, kilovolt peak

Patients were allocated consecutively in a 1:1 alternating sequence to either the BCS + TARGIT-IORT or BCS-only group (first to BCS + TARGIT-IORT, second to BCS-only). Blinding of patients and clinicians was not feasible given the nature of intraoperative radiotherapy, in line with prior TARGIT-IORT trials (Fig. 1).

All patients underwent standard diagnostic assessment. Following wide local excision (WLE), all patients underwent whole-breast irradiation (WBI) with a tumor bed boost. The BCS + TARGIT-IORT group received the boost intraoperatively using the Intrabeam system, while the BCS-only group received an EBRT boost.

### Inclusion and exclusion criteria

To be eligible, patients had to be adult women with histologically confirmed invasive breast carcinoma who were deemed suitable for BCS and WBI. All participants were expected to comply with long-term follow-up of at least 10 years, in accordance with institutional guidelines.

Patients were required to have at least one clinicopathological feature associated with increased risk of local recurrence such as, but not limited to, grade III histology, axillary nodal involvement, estrogen receptor (ER) and/or progesterone receptor (PgR) negativity, lymphovascular invasion, lobular carcinoma (local institutional criterion for boost), or an extensive intraductal component (EIC). Patients who required a tumor bed boost or were not suitable for TARGIT-IORT as sole radiotherapy were included. Patients with tumors initially unsuitable for BCS but rendered resectable through neoadjuvant systemic therapy were also eligible.

The exclusion criteria were bilateral breast cancer at presentation, severe comorbid illness significantly limiting life expectancy, or prior malignancy unless the estimated 10-year relapse-free survival exceeded 90% (e.g., non-melanoma skin cancer). Tumors exceeding 4 cm were excluded because of the maximum 5 cm cavity size accommodated by the Intrabeam applicator. Patients requiring treatment for metachronous breast cancer were excluded from the study.

### TARGIT-IORT boost

Prior to each procedure, quality assurance (QA) verification of the intrabeam miniaturized X-ray source (XRS) output was performed using a PTW 23342 ionization chamber (IC) in conjunction with the system probe adjuster and IC holder. The measured exposure (in Roentgens) was converted to the absorbed dose in water using a conversion factor of *f* = 0.881 cGy/R. The standard protocol delivers a dose of 20 Gy to the applicator surface (0 mm depth), with treatment times ranging between 16 and 50 min, based on manufacturer-provided calibration depth–dose curves [10].

The iso-dose distribution was verified using a dedicated water phantom (supplied by the manufacturer) equipped with a PTW-34013 soft chamber. This phantom setup allowed the XRS to be mounted above the water bath, with the probe inserted and positioned incrementally in a three-dimensional space (*x*, *y*, and *z* axes) to verify the dose distribution and ensure precise alignment prior to clinical use.

For patients in the BCS-only group, the boost was delivered using a specific linear accelerator available at the treatment facility of choice.

### Blood samples

Blood sampling at 24 h was selected to assess early postoperative systemic immune response. Two 25 ml blood samples were drawn into tubes containing ethylenediamine tetra-acetic acid (EDTA), as an anticoagulant, from patients at two specified intervals: 1 h prior to BCS and 24 h post-BCS + TARGIT-IORT or BCS-only. All blood samples were centrifuged within 2 h of collection and plasma was stored at − 80 °C.

### Enzyme-linked immunosorbent assay (ELISA) technique and markers

Plates pre-coated with antibodies against analytes of interest were used (Elabscience, USA). For each well, 100 µL of control or diluted sample was added and incubated at 37 °C for 90 min. Liquid was removed and biotinylated antibody was added for 1 h at 37 °C. The plates were washed and incubated with horseradish peroxidase for 30 min (37 °C). After the final wash, 90 µL of the substrate was added and incubated in the dark for 15 min. Upon reaching optimal color development, 50 µL of stop solution was added and the absorbance was measured at 450 nm. The following analytes were measured: caspase-8 (CASP-8), PRF1, HMGB1, granzyme B (GZMB), Fas ligand/tumor necrosis factor ligand superfamily member 6 (FASL-NFSF6), calreticulin (CRT), TRAIL, toll-like receptor 4 (TLR4), receptor for advanced glycation end products (RAGE), growth differentiation factor-15 (GDF15), and M-CSF.

Quantification was achieved by comparing the sample absorbance with a standard curve generated using serial dilutions of known standards. Each point was assayed in duplicate. A best-fit curve was constructed from the mean values.

### Data analysis

Continuous variables were summarized using means with standard deviations or medians with interquartile ranges, depending on distributional characteristics. Categorical variables were reported as frequencies and percentages. Baseline characteristics between patients receiving TARGIT-IORT + BCS and those BCS-only were compared using Welch’s t test for normally distributed continuous variables and the *χ*^2^ test for categorical variables. Blood plasma biomarker concentrations were analyzed in two steps. Within-group perioperative changes (pre-operative vs 24 h postoperative) were evaluated using the paired Wilcoxon signed-rank test, reflecting the non-parametric distribution of immune markers. Between-group differences in Δ values (post–pre) were assessed using the Wilcoxon rank-sum (Mann–Whitney *U*) test to determine whether TARGIT-IORT elicited immunogenic shifts distinct from surgery alone. Ninety-five percent confidence intervals were calculated for all biomarker means to demonstrate the dispersion of estimates. No imputation was performed for missing data, and all analyses were two-sided with a significance threshold of *p* < 0.05. Statistical analyses were conducted exclusively to evaluate biological response patterns and were not powered for survival outcomes given the exploratory nature of this translational cohort. Statistical analyses were performed using StataNow 18 SE (StataCorp, Texas USA).

## Results

### Patient characteristics

Forty-two patients were enrolled between September 2021 and July 2022. Twenty-six patients were enrolled in the TARGIT-IORT + BCS group receiving TARGIT-IORT as a boost, and 16 in the BCS-only group receiving EBRT boost per their existing regimen. Patient characteristics are presented in Table 1. The median age was significantly higher in the IORT group (55.9 years, range 51–61] years) than in the BCS-only group only group (48.4 years, range 42–55] years; *p* = 0.025). Treatment groups had significant difference between cancer grades (*p* = 0.02). The racial distribution did not differ between arms (white 77% vs. 81%, black 8% vs. 13%; overall *p* = 0.619). Five patients in the BCS-only group withdrew from the study, and blood samples from an additional five patients were compromised due to improper handling prior to cryostorage. Seroma formation was significantly higher in the BCS-only group (87% vs. 50%, *p* = 0.014), whereas fat necrosis was more common in the TARGIT-IORT + BCS group (27% vs. 6%), highlighting the different complication profiles between the boost modalities (Table 2).

**Table 1:** Patient demographics

	TARGIT-IORT + BCS	BCS-only	p value
*Patients*	26 (62%)	16 (38%)	
*Median age (range)*	55.9 years (51–61)	48.4 years (42–55)	0.0253*
*Race*			0.619**
White	20 (77%)	13 (81%)	
Black	2 (8%)	2 (13%)	
Asian	4 (15%)	1 (6%)	
*Menopausal status*			0.735**
Pre	10 (38%)	7 (44%)	
Post	16 (62%)	9 (56%)	
*Histology*			0.256**
IDC	24 (92%)	16 (100%)	
ILC	2 (8%)	0 (0%)	
*Side*			0.808**
Left	12 (46%)	8 (50%)	
Right	14 (54%)	8 (50%)	
*ER (X/8)*			0.653**
Positive	18 (69%)	10 (63%)	
Negative	8 (31%)	6 (37%)	
*PR (X/8)*			0.694**
Positive	13 (50%)	9 (56%)	
Negative	13 (50%)	7 (44%)	
*HER2*			0.636**
Positive	10 (38%)	5 (31%)	
Negative	16 (52%)	11 (69%)	
*KI67*			0.225**
< 10%	0 (0%)	1 (6%)	
10–20%	9 (35%)	0 (0%)	
> 20%	17 (65%)	15 (94%)	
*DCIS*			0.627**
Yes	11 (42%)	8 (50%)	
No	15 (58%)	8 (50%)	
*DCIS Grade*			0.144**
High	5 (19%)	7 (44%)	
Intermediate	6 (23%)	1 (6%)	
NA	15 (58%)	8 (50%)	
*Number of nodes removed*			0.407**
< 3	11 (42%)	9 (56%)	
3–5	13 (50%)	7 (44%)	
> 5	2 (8%)	0 (0%)	
*Number of nodes positive*			0.672**
0	13 (50%)	6 (37%)	
1	9 (35%)	8 (50%)	
2	3 (11%)	2 (13%)	
3	1 (4%)	0 (0%)	
*Extra nodal spread*			0.926**
Yes	23 (89%)	14 (87%)	
No	3 (11%)	2 (13%)	
*Grade*			0.02**
1	2 (8%)	1 (6%)	
2	14 (54%)	2 (13%)	
3	10 (38%)	13 (81%)	
*Peri neural invasion*			0.375**
Yes	4 (15%)	1 (6%)	
No	22 (85%)	15 (94%)	
*Vascular invasion*			0.763**
Yes	7 (27%)	5 (31%)	
No	19 (73%)	11 (69%)	
*Tumor size mm (range)*	28.2 (22.2–34)	32.2 (21–44)	0.762*
*Clinical T stage*			0.485**
1b	0 (0%)	1 (6%)	
1c	6 (23%)	2 (12.5%)	
2	18 (69%)	11 (69%)	
3	2 (8%)	2 (12.5%)	
*Clinical N stage*			0.832**
0	12 (46%)	6 (37%)	
1	13 (50%)	9 (56%)	
2	1 (4%)	1 (6%)	
*Pathological T stage*			0.436**
0	8 (31%)	7 (44%)	
1a	3 (11%)	1 (6%)	
1b	2 (8%)	3 (19%)	
1c	6 (23%)	3 (19%)	
2	5 (19%)	1 (6%)	
3	0 (0%)	1 (6%)	
Tis	2 (8%)	0 (0%)	
*Pathological N stage*			0.602**
0	19 (73%)	11 (69%)	
0i +	1 (4%)	0 (0%)	
1	5 (19%)	5 (31%)	
1mi	1 (4%)	0 (0%)	
*Pathological stage*			0.707**
0	10 (38%)	7 (44%)	
IA	7 (27%)	4 (25%)	
IB	1 (4%)	0 (0%)	
IIA	7 (27%)	4 (25%)	
IIB	1 (4%)	0 (0%)	
IIIA	0 (0%)	1 (6%)	
*PCR*			0.488**
Yes	8 (31%)	7 (44%)	
No	11 (42%)	7 (44%)	
NA	7 (27%)	2 (13%)	
*Margins*			1**
Positive	0 (0%)	0 (0%)	
Negative	26 (100%)	16 (100%)	
*Molecular subtype*			0.665**
Basal type	3 (11%)	4 (25%)	
HER enriched	9 (35%)	4 (25%)	
Luminal A	1 (4%)	1 (6%)	
Luminal B	13 (50%)	7 (44%)	

IDC, invasive ductal carcinoma; ILC, invasive lobular carcinoma; DCIS, ductal carcinoma in situ; IORT, intraoperative radiotherapy; EBRT, external beam radiotherapy; SLNB, sentinel lymph node biopsy; IDC, invasive ductal carcinoma; ILC, invasive lobular carcinoma; ER, estrogen receptor; PR, progesterone receptor; HER2, human epidermal growth factor receptor 2; Ki67, Ki-67 antigen; DCIS, ductal carcinoma in situ; NA, not applicable; PCR, pathologic complete response; Tis, tumor in situ; 0i +, isolated tumor cells in node; 1mi, micrometastasis in one lymph node; mm, millimeters; p, probability

*Welch *t* test

**Chi-squared test

**Table 2:** Surgery and complication rates

	TARGIT-IORT + BCS	BCS-only	p value
*Surgery type*			1**
Partial mastectomy and SLNB	26 (100%)	16 (100%)	
*Surgery complications*			1**
None	26 (100%)	16 (100%)	
*Oncotype*			0.606**
17	1 (4%)	0 (0%)	
18	1 (4%)	0 (0%)	
29	1 (4%)	0 (0%)	
39	1 (4%)	0 (0%)	
Not done	22 (84.8%)	16 (100%)	
*Chemotherapy*			0.536**
Adjuvant	3 (11%)	1 (6%)	
Neo-adjuvant	19 (73%)	14 (87%)	
None	4 (15%)	1 (6%)	
*Seroma*			0.014**
Yes	13 (50%)	14 (87%)	
No	13 (50%)	2 (13%)	
*Pneumonitis*			0.164**
Yes	1 (4%)	1 (6%)	
No	25 (96%)	15 (94%)	
*Late complications*			0.013**
Fat necrosis	7 (27%)	1 (6%)	
Fat necrosis and edema	0 (0%)	2 (13%)	
Hyper-pigmentation skin	2 (8%)	4 (25%)	
None	17 (65%)	9 (56%)	

IORT, intraoperative radiotherapy; EBRT, external beam radiotherapy; SLNB, sentinel lymph node biopsy; oncotype, oncotype DX recurrence score; adjuvant, adjuvant chemotherapy; neo-adjuvant, neoadjuvant chemotherapy; mm, millimeters; *p*, probability

**Chi-squared test

### ICD markers

Seven ICD-associated markers were quantified at baseline (1 h pre-surgery) and 24 h post-surgery across both groups (Tables 3, 4, 5; Fig. 1). As shown in Table 5, perioperative changes were analyzed using median values due to skewed biomarker distributions. In the TARGIT-IORT + BCS group, PRF1 declined from a median of 4.5–2.9 ng/ml (Wilcoxon *z* = – 2.229; *p* = 0.026), with a comparable reduction after BCS alone (2.8–2.0 ng/ml; *z* = – 2.556; *p* = 0.011; Δ *p* = 0.955).

**Table 3:** Blood plasma concentration pre-surgery and post-surgery + TARGIT-IORT

Plasma marker	Mean	SD	95 CI-	95 Ci +	Min	25th percentile	Median	75th percentile	Max
*TARGIT-IORT—pre-surgery*									
TLR4 (ng/ml)	1562.9	406.0	1391.5	1734.4	941.8	1328.1	1456.3	1600.1	3060.7
RAGE (pg/ml)	7037.1	3288.0	5648.7	8425.5	2411.2	4338.7	6768.9	8346.4	15,478.1
GDF15 (pg/ml)	2821.0	1202.9	2313.0	3328.9	1311.3	2111.9	2525.7	3438.5	6409.0
PRF1 (ng/ml)	5.0	2.8	3.8	6.2	1.1	3.0	4.5	6.3	11.4
HMGB1 (pg/ml)	4150.2	3623.9	2620.0	5680.5	823.3	1998.0	3410.5	5229.3	14,559.3
GZMB(pg/ml)	35.5	17.9	27.9	43.0	25.7	29.2	32.1	33.8	118.3
M-CSF (pg/ml)	301.6	116.0	252.6	350.6	69.3	227.7	266.7	338.0	584.9
CRT (ng/ml)	2119.0	1435.3	1513.0	2725.1	800.6	1305.4	1709.3	2307.6	7071.2
TRAIL (pg/ml)	235.0	43.8	216.5	253.5	123.4	214.6	246.1	259.9	311.5
FASL-NFSF6 (pg/ml)	1110.3	223.2	1016.0	1204.6	718.7	933.0	1087.1	1252.0	2147.8
CASP-8 (ng/ml)	704.2	344.7	558.6	849.7	418.0	521.0	557.2	683.7	1700.8
*TARGIT-IORT—24 h post-surgery*									
TLR4 (ng/ml)	1321.8	292.8	1198.2	1445.4	840.6	1114.5	1331.3	1434.7	2298.8
RAGE (pg/ml)	6709.3	6568.0	3935.9	9482.7	1700.5	3890.8	4498.9	7083.9	33,175.0
GDF15 (pg/ml)	2844.3	1524.1	2200.7	3487.8	977.8	1918.7	2481.6	3133.5	7170.0
PRF1 (ng/ml)	3.3	1.9	2.5	4.1	0.6	2.0	2.9	4.9	7.5
HMGB1 (pg/ml)	340.7	196.7	257.6	423.8	88.5	157.0	359.6	421.4	783.7
GZMB(pg/ml)	39.8	45.4	20.6	59.0	26.3	28.6	30.3	32.7	252.6
M-CSF (pg/ml)	402.4	179.9	326.4	478.4	149.8	275.9	386.7	479.1	924.3
CRT (ng/ml)	1681.6	729.8	1373.4	1989.8	769.5	1157.9	1364.4	2249.0	3724.3
TRAIL (pg/ml)	169.5	59.3	144.5	194.6	79.3	128.7	155.4	190.4	320.1
FASL-NFSF6 (pg/ml)	890.2	245.3	786.6	993.8	511.8	736.3	839.3	1102.1	1351.1
CASP-8 (ng/ml)	710.3	574.7	467.7	953.0	411.9	460.5	539.1	695.7	3204.8

TARGIT-IORT, targeted intraoperative radiotherapy; TRL-4, toll-like receptor 4; RAGE, receptor for advanced glycation end products; GDF15, growth differentiation factor-15; PRF1, perforin-1; HMGB1, high-mobility group box 1; GZMB, granzyme B; M-CSF, macrophage colony-stimulating factor; CRT, calreticulin; TRAIL, TNF-related apoptosis-inducing ligand; FASL-NFSF6, Fas ligand (TNF superfamily member 6); CASP-8, Caspase-8; SD, standard deviation; CI, confidence interval

**Table 4:** Blood plasma concentration pre-surgery and 24 h post-surgery

Plasma marker	Mean	SD	95 CI−	95 CI +	Min	25th percentile	Median	75th percentile	Max
*BCS-only—pre-surgery*									
PRF1	4.5	4.3	2.2	6.9	1.2	2.0	2.8	4.8	15.6
HMGB1	994.4	470.1	734.1	1254.7	165.2	700.6	1040.7	1269.2	2981.0
GZMB	31.7	2.9	30.1	33.3	27.4	29.2	31.0	32.7	132.2
CRT	1921.7	1570.8	1051.8	2791.6	763.3	955.6	1259.9	2378.1	5782.5
TRAIL	212.4	40.0	190.3	234.6	163.9	178.2	209.7	252.7	298.4
FASL-NFSF6	1151.3	521.2	862.7	1439.9	628.1	860.8	991.4	1267.2	2515.6
CASP-8	580.2	153.0	495.5	664.9	45.8	487.7	539.1	635.5	887.6
TRL-4	1166.1	264.9	1019.4	1312.9	813.4	932.3	1173.7	1406.9	1624.7
RAGE	17,185.6	11,810.8	10,644.9	23,726.2	2925.3	8571.4	17,303.4	21,152.9	46,027.8
GDF15	2590.8	1603.0	1703.1	3478.5	1252.6	1613.5	1933.9	3484.8	7170.0
M-CSF	269.3	109.8	208.5	330.1	91.1	188.3	259.3	289.7	504.1
*BCS-only—24 h post-surgery*									
PRF1	2.8	3.6	0.9	4.8	0.3	0.8	2.0	3.3	14.4
HMGB1	311.8	211.2	194.8	428.8	95.8	127.9	263.6	469.6	752.3
GZMB	38.5	26.4	23.9	53.1	27.4	29.2	31.0	32.7	132.2
CRT	2174.5	2358.3	868.5	3480.5	763.3	955.5	1260.0	2378.1	5782.5
TRAIL	291.9	475.2	28.7	555.1	107.4	125.0	154.3	232.4	1999.9
FASL-NFSF6	987.3	480.3	721.4	1253.3	518.5	728.6	782.3	1199.2	2164.3
CASP-8	668.7	265.4	521.7	815.6	454.4	472.6	514.9	821.7	1283.2
TRL-4	1195.7	396.4	976.2	1415.3	646.9	913.4	1225.7	1311.0	2243.3
RAGE	15,990.6	14,549.4	7933.4	24,047.8	2677.1	5872.3	11,534.3	16,463.8	49,159.6
GDF15	2379.2	1014.8	1817.3	2941.2	1332.9	1627.8	2049.3	2885.8	4867.2
M-CSF	326.6	267.1	178.7	474.5	111.2	172.1	290.8	369.5	1229.8

TARGIT-IORT, targeted intraoperative radiotherapy; PRF1, perforin-1; HMGB1, high-mobility group box 1; GZMB, granzyme B; CRT, calreticulin; TRAIL, TNF-related apoptosis-inducing ligand; FASL-NFSF6, Fas ligand (TNF superfamily member 6); CASP-8, caspase-8; SD, standard deviation; CI, confidence interval

**Table 5:** The pre- to postoperative changes in circulating immune biomarkers in patients treated with TARGIT-IORT plus breast-conserving surgery compared with those receiving BCS alone

Biomarker	Group	*n*		Pre-median (IQR)		Post-median (IQR)		Wilcoxon *z*		*p*-value (within group^1^)		Δ *p*-value (Wilcoxon rank-sum/Mann–Whitney *U*) (TARGIT-IORT + BCS vs BCS only^2^)
TLR4 (ng/ml)	TARGIT-IORT + BCS	26	1456.3		1331.3		− 2.714		0.007		0.421	
TLR4 (ng/ml)	BCS-only	16	1173.7		1225.7		− 1.193		0.233			
RAGE (pg/ml)	TARGIT-IORT + BCS	26	6768.9		4499.0		− 1.200		0.230		0.458	
RAGE (pg/ml)	BCS-only	16	17,303.4		11,534.3		− 0.568		0.570			
GDF15 (pg/ml)	TARGIT-IORT + BCS	26	2525.7		2481.6		− 0.029		0.977		0.627	
GDF15 (pg/ml)	BCS-only	16	1933.9		2049.3		0.057		0.955			
PRF1 (ng/ml)	TARGIT-IORT + BCS	26	4.5		2.9		− 2.229		0.026		0.955	
PRF1 (ng/ml)	BCS-only	16	2.8		2.0		− 2.556		0.011			
HMGB1 (pg/ml)	TARGIT-IORT + BCS	26	3410.5		359.8		− 4.286		< 0.001		< 0.001	
HMGB1 (pg/ml)	BCS-only	16	1040.7		263.6		− 3.351		< 0.001			
GZMB (pg/ml)	TARGIT-IORT + BCS	26	32.1		30.3		− 1.615		0.106		0.100	
GZMB (pg/ml)	BCS-only	16	31.5		30.9		− 0.400		0.689			
M-CSF (pg/ml)	TARGIT-IORT + BCS	26	266.7		386.7		2.086		0.037		0.049	
M-CSF (pg/ml)	BCS-only	16	259.3		290.8		0.625		0.532			
CRT (ng/ml)	TARGIT-IORT + BCS	26	1709.3		1364.4		− 1.257		0.209		0.392	
CRT (ng/ml)	BCS-only	16	1260.0		1320.1		− 0.568		0.570			
TRAIL (pg/ml)	TARGIT-IORT + BCS	26	246.1		155.5		− 3.314		0.001		0.189	
TRAIL (pg/ml)	BCS-only	16	209.7		154.3		− 1.647		0.100			
FASL-NFSF6(pg/ml)	TARGIT-IORT + BCS	26	1087.1		839.3		− 3.000		0.003		0.458	
FASL-NFSF6(pg/ml)	BCS-only	16	991.4		782.3		− 1.533		0.125			
CASP-8 (ng/ml)	TARGIT-IORT + BCS	26	557.2		539.1		− 0.671		0.502		0.098	
CASP-8 (ng/ml)	BCS-only	16	539.1		514.9		1.224		0.221			

For each biomarker, within-group changes were assessed using the paired Wilcoxon signed-rank test, and between-group differences in delta values (post–pre) were compared using the Wilcoxon rank-sum (Mann–Whitney *U*) test

IORT, intraoperative radiotherapy; BCS, breast-conserving surgery; TLR4, toll-like receptor 4; RAGE, receptor for advanced glycation end products; GDF15, growth differentiation factor-15; PRF1, perforin-1; HMGB1, high-mobility group box 1; GZMB, granzyme B; M-CSF, macrophage colony-stimulating factor; CRT, calreticulin; TRAIL, TNF-related apoptosis-inducing ligand; FASL-NFSF6, Fas ligand tumor necrosis factor (TNF superfamily member 6; CASP-8, caspase-8; Δ p-value, p-value for between-group comparison of the change (post–pre)

^1^Within-group p-values represent the comparison of pre- and postoperative biomarker levels within each treatment group, assessed using the paired Wilcoxon signed-rank test

^2^Between-group Δ p-values represent the comparison of the change in biomarker levels (post–pre) between the TARGIT-IORT + BCS and BCS-only groups, assessed using the Wilcoxon rank-sum (Mann–Whitney *U*) test

HMGB1, which exhibited the greatest variability by mean levels in Table 3 (mean 4150.2 pg/ml pre-op vs 340.7 pg/ml post-op), also showed the most pronounced median decrease in Table 5, falling from 3410.5 to 359.8 pg/ml after TARGIT-IORT (*z* = – 4.286; *p* < 0.001). BCS-only group alone produced a smaller but still significant decline (1040.7–263.6 pg/ml; *z* = – 3.351; *p* < 0.001), with a statistically greater Δ reduction in the TARGIT-IORT group (Δ *p* < 0.001).

Death ligand changes were similarly aligned across tables. TRAIL, with mean levels in Table 3 decreasing from 235.0 to 169.5 pg/ml, also showed a significant median decline in Table 5 (246.1–155.5 pg/ml; *z* = – 3.314; *p* = 0.001). BCS-only group changes were modest and non-significant (209.7–154.3 pg/ml; *p* = 0.100). FASL-NFSF6 decreased significantly after TARGIT-IORT + BCS (1087.1–839.3 pg/ml; *z* = – 3.000; *p* = 0.003) but not following BCS alone (991.4–782.3 pg/ml; *p* = 0.125), though the Δ comparison was not statistically different (Δ *p* = 0.458).

Other ICD mediators, including CRT, GZMB, and CASP-8, showed only small median shifts in Table 5 (all within-group *p* ≥ 0.10), consistent with their mean values in Tables 3 and 4, which also demonstrated limited perioperative movement.

### Cytokine and damage-associated molecular patterns DAMP profiles

**Fig. 2:**
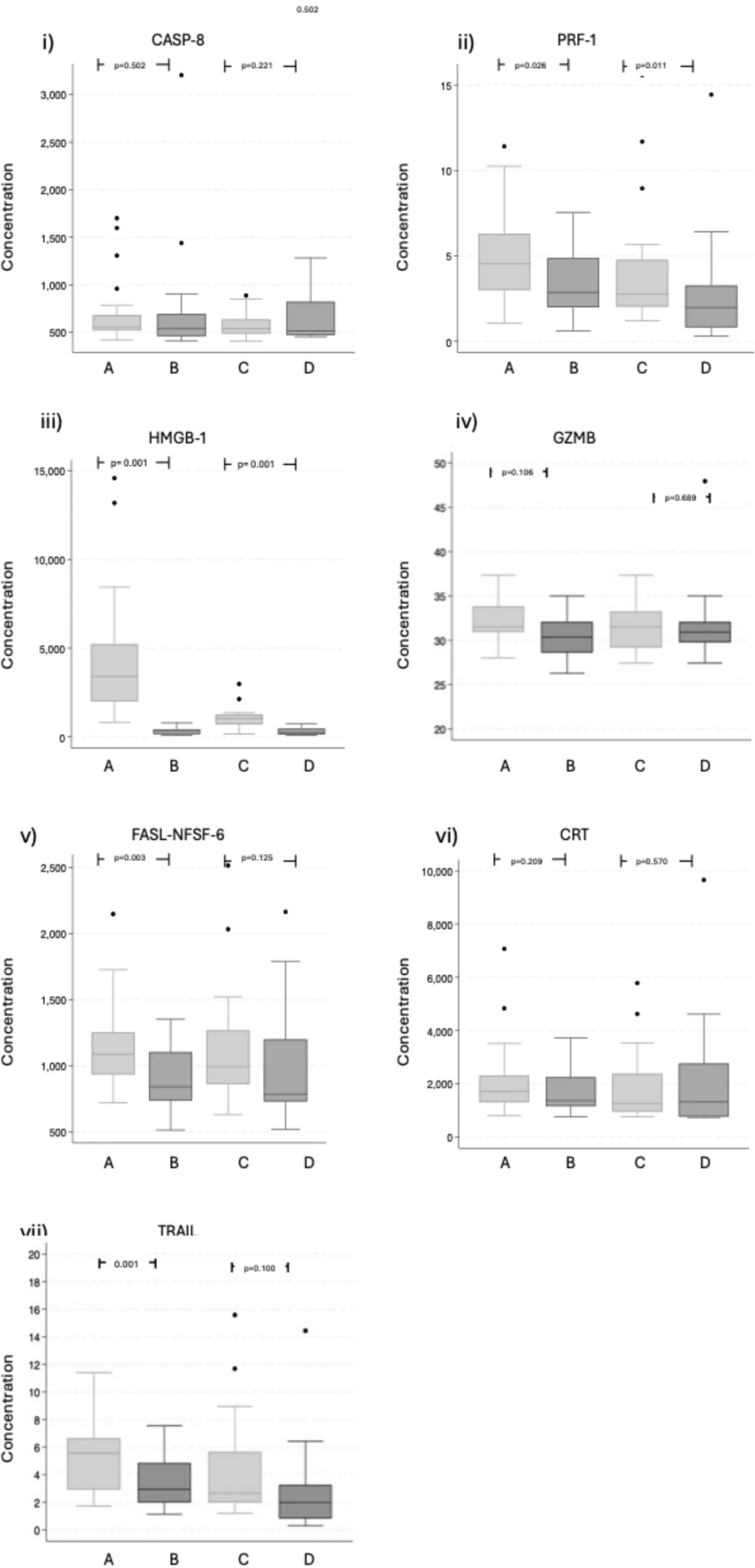
Comparative expression of immunogenic cell death and immune markers in blood plasma following breast cancer treatment: this figure illustrates the concentrations of seven immunogenic cell death (ICD) and immune-related markers in blood plasma across four clinical groups: **A** pre-surgery targeted intraoperative radiotherapy (TARGIT-IORT), **B** 24 h post-surgery TARGIT-IORT, **C** pre-surgery BCS-only group, and D 24 h post-surgery BCS-only group. The markers assessed include caspase-8 (CASP-8, i), perforin-1 (PRF1, ii), high-mobility group box 1 (HMGB1, iii), granzyme B (GZMB, iv), Fas ligand/tumor necrosis factor ligand superfamily member 6 (FASL-NFSF6, v), calreticulin (CRT, vi), and tumor necrosis factor-related apoptosis-inducing ligand (TRAIL, vii). The solid horizontal line within each box represents the median. Boxes indicate the interquartile range (25th–75th percentile), whiskers represent the data range, and points denote outliers. Summary statistics in the tables are presented as means and therefore may not correspond directly to the medians shown in the boxplots

Four additional cytokine/DAMP mediators were assessed (Tables 3, 4, 5; Fig. 2). M-CSF, which increased from a mean of 301.6–402.4 pg/ml in Table 3, showed a corresponding significant median rise from 266.7 to 386.7 pg/ml after TARGIT-IORT + BCS in Table 5 (*z* = 2.086; *p* = 0.037). No significant change was observed after BCS alone (259.3–290.8 pg/ml; *p* = 0.532), resulting in a significant Δ difference (Δ *p* = 0.049).

**Fig. 3:**
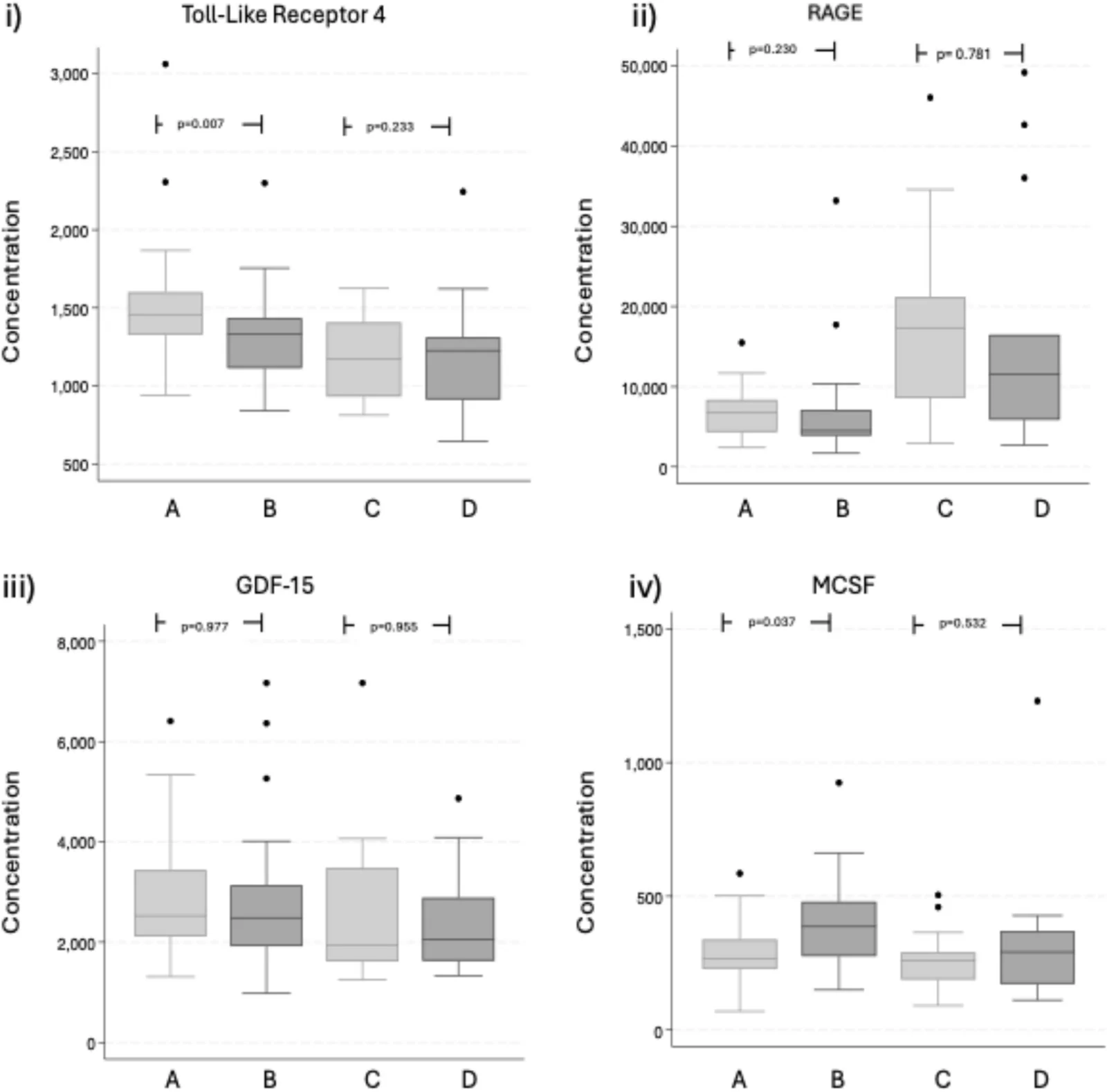
Expression of inflammatory and damage-associated immune markers in blood plasma following breast cancer radiotherapy: this figure presents boxplots of four inflammatory and damage-associated molecular pattern (DAMP) markers measured in blood plasma across four patient groups: **A** pre-surgery targeted intraoperative radiotherapy (TARGIT-IORT), **B** 24 h post-surgery TARGIT-IORT, **C** pre-surgery BCS-only group, and D 24 h post-surgery BCS-only group. The markers include Toll-like receptor 4 (TLR4, i), receptor for advanced glycation end products (RAGE, ii) growth differentiation factor-15 (GDF15, iii), and macrophage colony-stimulating factor (M-CSF, iv). These immune mediators reflect the innate immune response and tissue injury signaling within the postoperative tumor bed. The solid horizontal line within each box represents the median. Boxes indicate the interquartile range (25th–75th percentile), whiskers represent the data range, and points denote outliers. Summary statistics in the tables are presented as means and therefore may not correspond directly to the medians shown in the boxplots

TLR4 also demonstrated concordant findings across tables: mean levels fell from 1562.9 to 1321.8 ng/ml in Table 3, and median levels decreased significantly in Table 5 (1456.3–1331.3 ng/ml; *z* = – 2.714; *p* = 0.007). BCS-only group values showed a small, non-significant rise (1173.7–1225.7 ng/ml; *p* = 0.233; Δ *p* = 0.421) (Fig. 3).

In contrast, RAGE and GDF15 showed wide inter-individual variability on mean values (Tables 3, 4) and no significant perioperative median changes (RAGE *p* = 0.230 and *p* = 0.570; GDF15 *p* = 0.977 and *p* = 0.955 for TARGIT-IORT and BCS-only, respectively), with all Δ values non-significant (Δ *p* ≥ 0.458).

## Discussion

### TARGIT-IORT recent biological effects

Emerging translational data further support that TARGIT-IORT/IORT alters the tumor microenvironment (TME) by triggering ICD, a form of regulated cell death that not only eliminates tumor cells but also activates the immune system response [2, 11]. During ICD, dying tumor cells undergo cellular stress responses that cause redistribution (within the cell) and release (into the surrounding environment) of specific molecules called DAMPs, such as HMGB1, calreticulin, and adenosine triphosphate (ATP). Recent single-cell RNA sequencing analyses demonstrate that IORT combined with surgery reshapes the peripheral immune landscape enhancing cytotoxic activity of cluster of differentiation-8 (CD8⁺) effector T cells, and significant T-cell clonal expansion, alongside increased responsiveness to PD-1 blockade, suggesting potential synergy with immunotherapy [3]. At the tumor bed level, transcriptomic analyses reveal enrichment of inflammatory and T-cell activation pathways together with enhanced antigen presentation signaling, and alterations in wound fluid cytokine composition and suppression of mesenchymal stromal cell activity indicate a microenvironment less permissive to tumor cell proliferation and invasion [5, 12]. In parallel, radiotherapy-induced modulation of immune checkpoint signaling, including reductions in soluble PD-1 and CTLA-4, provided further mechanistic rationale for enhanced immune recognition and potential combinatorial strategies with checkpoint inhibitors [13]. Collectively, these findings support the concept that TARGIT-IORT acts as a perioperative immunobiological modifier, although the clinical significance and durability of these effects remain to be defined.

The updated 2024 ASTRO guidelines now recommend against IORT as sole clinical radiation therapy outside of prospective trials or registries, citing concerns over higher recurrence rates and technical limitations [4]. The Society of Surgical Oncology and ASTRO emphasize careful patient selection and further recommend IORT only within research settings [4]. Some international guidelines still include IORT for select patients, but US consensus is increasingly restrictive [4]. Hence, results should be interpreted within a translational framework rather than as evidence for therapeutic efficacy.

### Patient demographics and baseline imbalances

This pilot cohort consisted of 42 participants, with 26 (62%) assigned to the BCS + TARGIT-IORT group and 16 (38%) to the BCS-only group. Despite broadly similar clinical and pathological characteristics across most variables, the two cohorts were not fully comparable: the IORT group tended to be older (55.9 years vs 48.4 years; *p* = 0.025), while the BCS-only group contained a disproportionately high number of grade 3 tumors (81% vs 38%; *p* = 0.020). These differences emerged in the context of a small study population and the alternating, non-random method of allocating patients, both of which naturally increase the likelihood of imbalances.

The 24 h time point was selected to capture a more stable and integrated systemic immune response, as the postoperative host response reflects a coordinated cascade of DAMPs and downstream inflammatory mediators that consolidate over the first postoperative day, whereas earlier time points are dominated by rapid, transient, and highly variable perioperative fluctuations [14].

Given the limited number of evaluable patients, breaking the dataset into smaller subgroups, such as by age, would have produced groups too small to yield any credible or interpretable comparisons and was therefore not pursued. Instead, the analyses were restricted to whole-cohort comparisons appropriate for an exploratory mechanistic study. Overall, the demographic and pathological disparities observed here should be interpreted as inherent limitations of a preliminary translational investigation rather than indicators of systematic bias.

### Immunogenic cell death and innate immune recognition pathways

Across the seven ICD-related biomarkers analyzed (Table 5; Fig. 1), TARGIT-IORT produced a rapid, treatment-specific shift in perioperative immune signaling that was not mirrored in the BCS-only cohort. The clearest and most biologically meaningful change was the profound decline in circulating HMGB1, which fell from a median of 3410.5–359.8 pg/ml within 24 h of TARGIT-IORT (Wilcoxon *z* = – 4.286; *p* < 0.001). Although HMGB1 also decreased following BCS alone (*p* < 0.001), the between-group Δ was significantly greater after TARGIT-IORT (Δ *p* < 0.001), demonstrating that intraoperative irradiation induces a substantially more profound HMGB1 response. The marked decline in circulating high-mobility group box 1 protein (HMGB1) within 24 h of intraoperative irradiation in breast cancer is consistent with rapid engagement of immunogenic cell death pathways, particularly the HMGB1–TLR4 axis, which is central to dendritic cell activation and cross-presentation of tumor antigens to CD8^+^ T cells. The clinical relevance of this pathway is underscored by genetic studies showing that breast cancer patients carrying the TLR4 Asp299Gly loss-of-function polymorphism experience significantly earlier relapse after anthracycline-based chemotherapy and radiotherapy compared to those with wild-type TLR4 [15, 16]. This polymorphism impairs HMGB1 binding to TLR4, disrupting dendritic cell activation and antigen cross-presentation. The rapid decline in circulating HMGB1 within 24 h post-IORT, absent in surgery-only patients, strongly suggests active receptor–ligand engagement and internalization rather than simple clearance. Upon binding HMGB1, TLR4 undergoes endocytosis along with its ligand, initiating Myeloid Differentiation Primary Response 88 (MyD88)-dependent signaling cascade that activate Nuclear Factor kappa-light-chain-enhancer of activated B cells (NF-κB) which promote dendritic cell maturation [17, 18]. Surgical trauma is known to induce HMGB1 release peaking on postoperative day 3, while in-vitro studies show that radiation-induced HMGB1 release occurs later (typically 72–96 h), reflecting the time required for radiation-induced cell death to manifest and for dying cells to release nuclear HMGB1 [19–21]. Importantly, extracellular HMGB1 has a very short functional half-life due to rapid receptor-mediated internalization, oxidation, and active uptake by immune cells, resulting in substantial depletion from circulation within hours [22–24]. In this context, the key distinguishing feature is the concurrent decline in TLR4 observed following TARGIT-IORT but not in surgery-only patients, which supports active HMGB1–TLR4 engagement and receptor internalization within the irradiated microenvironment and the 24 h timepoint may be too early to capture HMGB1 release [9, 16, 18].

The decline in circulating TRAIL is consistent with ligand–receptor binding followed by rapid internalization. TRAIL decreased markedly from 246.1 to 155.5 pg/ml in the TARGIT-IORT cohort (*p* = 0.001), whereas the BCS-only group demonstrated a non-significant decline. FASL-NFSF6 similarly fell after TARGIT-IORT (1087.1 → 839.3 pg/ml; *p* = 0.003), although the between-group Δ effect did not reach significance. Upon TRAIL binding, death receptor 4 (DR4) and death receptor 5 (DR5) undergo rapid clathrin-dependent endocytosis in a concentration- and time-dependent manner, sensitizing them to apoptosis via their respective ligands (FasL) [24]. This internalization occurs within minutes of ligand exposure and is followed by receptor recycling over 4–6 h [24]. Importantly, radiation upregulates DR5 expression on tumor cells in a p53-dependent manner, creating more binding sites for TRAIL and accelerating ligand consumption [26]. The synergistic effect of radiation and TRAIL on tumor cell killing is well documented, with radiation sensitizing cells to TRAIL-induced apoptosis through enhanced death receptor expression and improved death-inducing signaling complex (DISC) formation [27]. This study supports that radiation induces DR4/DR5 upregulation within hours, and TRAIL binding triggers rapid receptor–ligand complex internalization [25]. In breast cancer cells specifically, TRAIL induces rapid endocytosis of DR4 and DR5, with internalized DR4 undergoing caspase-dependent cleavage [28]. This consumption of circulating TRAIL through binding to upregulated death receptors on irradiated tumor cells would manifest as a decline in plasma TRAIL levels. The activation of DR4/DR5 pathways is central to the immunogenic and apoptotic effects of radiotherapy, contributing to tumor cell clearance and modulation of the local immune response consistent with ligand consumption.

The reduction in circulating FasL likely reflects engagement with Fas (cluster of differentiation 95 (CD95)) receptors on target cells. Critically, membrane-bound FasL (mFasL) is essential for Fas-induced apoptosis, whereas soluble FasL (sFasL) has over 1,000-fold reduced apoptotic activity [29, 30]. Accordingly, the observed decline in plasma FasL is most consistent with ligand utilization within active apoptotic signaling, potentially reflecting a combination of receptor binding and internalization, reduced shedding from the membrane-bound form due to ongoing target cell engagement, and consumption of sFasL through Fas interaction [29, 30]. This interpretation is further supported by the known effects of radiation, which upregulates Fas receptor expression on tumor cells in a p53-dependent manner, thereby increasing available binding sites for FasL [32], while death receptor pathways (Fas, TRAIL, TNF-α) are activated in a temporally coordinated manner following irradiation to mediate both direct and bystander cytotoxic effects [33].

The decline in circulating PRF1 is consistent with early cytotoxic lymphocyte activation, degranulation, and granule exocytosis. Upon target cell recognition, PRF1-containing granules are rapidly released into the immunological synapse, with exocytosis occurring within seconds of calcium influx, enabling pore formation on target cell membranes and facilitating granzyme delivery despite rapid membrane repair [34]. Experimental studies demonstrate that PRF1 protein levels decrease immediately following effector–target interaction, reflecting granule release and utilization, followed by recovery through transcriptional reactivation and granule refilling during serial killing [35, 36]. This dynamic process includes rapid mRNA turnover and resynthesis, allowing cytotoxic T cells to sustain repeated effector function. Accordingly, the observed reduction in circulating PRF1 after TARGIT-IORT most plausibly reflects active effector cell engagement and perforin utilization within the tumor microenvironment, rather than diminished cytotoxic capacity, and is consistent with enhanced T-cell cytotoxicity observed [19].

M-CSF demonstrated a significant TARGIT-IORT-specific increase (*p* = 0.037), with no corresponding change following BCS alone and a significant between-group difference (Δ *p* = 0.049), supporting a radiation-driven effect rather than surgical stress alone. Mechanistically, this is consistent with radiation-induced DNA damage signaling, whereby activation of ABL Proto-Oncogene 1 (ABL1) enhances colony-stimulating factor 1 (CSF-1) transcription [37]. Within the broader biomarker profile, M-CSF elevation complements the concurrent decline in HMGB1, TLR4, TRAIL, FasL, and PRF1, suggesting a coordinated immune response characterized by early DAMP engagement and cytotoxic effector utilization, followed by recruitment of monocytes/macrophages to the irradiated tumor bed. Functionally, M-CSF may serve dual roles in this context, promoting antigen-presenting cell recruitment and amplification of immunogenic cell death-driven immune responses [38], while also contributing to wound healing and tissue remodeling through macrophage-mediated pathways [39]. The balance between these effects is likely governed by local cytokine conditions, with the concurrent ICD-associated signaling favoring an early pro-inflammatory (M1) phenotype [40]. Collectively, these findings further support the interpretation of TARGIT-IORT as a perioperative immunobiological modifier, inducing coordinated activation of innate sensing, cytotoxic effector pathways, and subsequent immune cell recruitment.

Other mediators, GZMB, CRT, and CASP-8, did not exhibit significant perioperative shifts, although trends in the TARGIT-IORT + BCS group indicate early downward movement. The absence of a statistically significant change may be attributable to timing, as CRT externalization and caspase-8 cleavage are transient events that likely peak earlier than the 24-h sampling window. Perioperative modulation of GZMB, CRT and CASP-8 reflects the tightly coordinated kinetics of ICD. Granzyme B typically rises within the first 24–48 h after irradiation, signaling rapid activation of cytotoxic CD8⁺ T cells and early effector engagement in tumor clearance [3]. CRT exposure follows a biphasic pattern, with an initial pre-apoptotic surge and a later phase as apoptosis progresses, functioning as a critical “eat-me” signal that promotes dendritic cell phagocytosis and antigen presentation [41]. This process is orchestrated by endoplasmic reticulum stress and requires early CASP-8 activation, as CASP-8 cleavage precedes and drives calreticulin translocation and is essential for the immunogenicity of apoptosis [41]. Because these events unfold within hours, sampling restricted to a single 24 h postoperative time point is unlikely to capture the full temporal dynamics. Earlier and more frequent sampling intervals may be necessary in future studies to characterize accurately the biphasic behavior of CRT, the early activation peak of GZMB, and the initiating role of CASP-8 in the ICD cascade [42].

Overall, the biomarker patterns demonstrate that TARGIT-IORT triggers a rapid and coordinated ICD-associated immune response, marked by a profound decline in HMGB1 (3410.5 → 359.8 pg/ml; Δ*p* < 0.001), significant reductions in TRAIL (246.1 → 155.5 pg/ml; *p* = 0.001) and FASL (1087.1 → 839.3 pg/ml; *p* = 0.003), concurrent TLR4 engagement (1456.3 → 1331.3 ng/ml; *p* = 0.007), and early cytotoxic granule utilization reflected by PRF1 depletion (4.5 → 2.9 ng/ml; *p* = 0.026). Taken together, these signals (spanning DAMP release, death receptor pathway activation, and cytotoxic effector deployment) indicate that TARGIT-IORT not only eradicates microscopic residual disease but also initiates immediate immune priming within the surgical bed. This mechanistic profile provides biologically plausible support for the durable clinical performance of TARGIT-IORT and highlights the importance of future studies incorporating multi-timepoint sampling and adequately powered randomized cohorts to fully define the immunological consequences of intraoperative radiotherapy in breast cancer.

### Limitations and future work

This study is limited by small sample size, unequal group distribution, and non-random allocation, all of which increase the risk of baseline imbalances and restrict the strength of causal inference. Attrition within the BCS-only arm further reduced evaluable numbers and limited the ability to perform subgroup analyses. Biomarker profiling relied solely on peripheral blood plasma, which may not reflect intratumoral immune activity, and sampling was confined to the immediate perioperative period, capturing early ICD-related changes but missing later adaptive and remodeling phases.

These limitations mean the findings should be regarded as hypothesis-generating. Future work should include adequately powered, randomized multicenter cohorts with improved biospecimen handling and extended longitudinal sampling, integrating tissue-level and single-cell analyses to validate the perioperative immune signatures observed here and to clarify their relevance for immunotherapy sequencing in breast cancer.

## Conclusion

In this exploratory translational study, TARGIT-IORT produced a distinct and rapidly evolving perioperative immune signature characterized by coordinated modulation of ICD-related biomarkers and selective activation of innate immune pathways. The marked early declines in HMGB1, TRAIL, FASL, PRF1, and TLR4, together with the rise in M-CSF, suggest that intraoperative irradiation not only sterilizes microscopic residual disease but also initiates immediate immune priming within the tumor bed at the time of surgical excision. Although limited by small sample size and short sampling intervals, these findings provide mechanistic insight into the biological effects of IORT and support further investigation of its immunomodulatory potential. Larger, randomised, multicentre studies with extended longitudinal and tissue-level immune profiling will be essential to validate these observations and determine their relevance for modern breast cancer radiotherapy and future combinatorial approaches with immunotherapy.
